# Drought-Induced Carbon and Water Use Efficiency Responses in Dryland Vegetation of Northern China

**DOI:** 10.3389/fpls.2019.00224

**Published:** 2019-02-26

**Authors:** Chengcheng Gang, Yi Zhang, Liang Guo, Xuerui Gao, Shouzhang Peng, Mingxun Chen, Zhongming Wen

**Affiliations:** ^1^Institute of Soil and Water Conservation, Northwest A&F University, Yangling, China; ^2^Institute of Soil and Water Conservation, Chinese Academy of Sciences and Ministry of Water Resources, Yangling, China; ^3^International Center for Climate and Global Change Research, School of Forestry and Wildlife Sciences, Auburn University, Auburn, AL, United States; ^4^College of Agronomy, Northwest A&F University, Yangling, China

**Keywords:** carbon use efficiency, drought severity index, dryland vegetation, northern China, water use efficiency

## Abstract

Given the context of global warming and the increasing frequency of extreme climate events, concerns have been raised by scientists, government, and the public regarding drought occurrence and its impacts, particularly in arid and semi-arid regions. In this paper, the drought conditions for the forest and grassland areas in the northern region of China were identified based on 12 years of satellite-based Drought Severity Index (DSI) data. The impact of drought on dryland vegetation in terms of carbon use efficiency (CUE) and water use efficiency (WUE) were also investigated by exploring their correlations with DSI. Results indicated that 49.90% of forest and grassland experienced a dry trend over this period. The most severe drought occurred in 2001. In general, most forests in the study regions experienced near normal and wet conditions during the 12 year period. However, grasslands experienced a widespread drought after 2006. The forest CUE values showed a fluctuation increase from 2000 to 2011, whereas the grassland CUE remained steady over this period. In contrast, WUE increased in both forest and grassland areas due to the increasing net primary productivity (NPP) and descending evapotranspiration (ET). The CUE and WUE values of forest areas were more sensitive to droughts when compared to the values for grassland areas. The correlation analysis demonstrated that areas of DSI that showed significant correlations with CUE and WUE were 17.24 and 10.37% of the vegetated areas, respectively. Overall, the carbon and water use of dryland forests was more affected by drought than that of dryland grasslands.

## Introduction

Recently, droughts have been frequently recorded due to climatic warming from elevated concentrations of greenhouse gasses. This warming exacerbates water resource stress and poses a significant threat to food security and the sustainability of human activities in these areas (Vorosmarty et al., 2000; Rosegrant et al., 2003). The reports of the Intergovernmental Panel on Climate Change suggest that drought frequency will likely increase by the end of 21st century, particularly in regions that are currently dry (IPCC, 2014). At the ecosystem scale, drought will reduce the carbon sequestration ability of vegetation and aggravate the water evaporation rate of ecosystems (Ciais et al., 2005; Zhao and Running, 2010). The ecosystem-scale carbon use efficiency (eCUE), which is defined as the ratio of net primary productivity (NPP) to gross primary productivity (GPP), describes the capacity of an ecosystem to transfer the carbon from the atmosphere to vegetation biomass (DeLucia et al., 2007). The ecosystem-scale water use efficiency (eWUE), a ratio of NPP to evapotranspiration (ET), measures the net carbon uptake per amount of water lost from the ecosystem (Beer et al., 2009; Gang et al., 2016a,b). The eCUE and eWUE are two vital indicators for addressing the ecosystem function in terms of carbon and water cycles (Webb et al., 1978; LeHouerou, 1984; DeLucia et al., 2007). The satellite-based imageries provide an effective way in monitoring drought severity and vegetation responses in term of carbon and water cycles (AghaKouchak et al., 2015; Liu Y. et al., 2015). The impacts of droughts on CUE and WUE have been widely reported in multiple scales (Webb et al., 1978; DeLucia et al., 2007; Liu Y. et al., 2015; Gang et al., 2016a). However, the occurrence of drought and its subsequent influences on ecosystem-scale CUE and WUE have not been extensively investigated, especially in the arid and semi-arid regions. Therefore, further research is needed on the extent and duration of droughts as well as their impacts on the carbon and water cycles of dryland vegetation.

Precipitation is a primary input data in most drought indices, which have been widely used in monitoring drought conditions at different levels, such as the Standardized Precipitation Index (SPI) (McKee et al., 1995), the Palmer Drought Severity Index (PDSI) (Palmer, 1965), the Temperature-Vegetation Dryness Index (TVDI) (Sandholt et al., 2002), the Vegetation, Water and Thermal Stress Index (VWTCI) (Shakya and Yamaguchi, 2010), and the Percentage of Precipitation Anomaly (PPA). The large-scale effects of drought on vegetation are detectable with the help of remote sensing technology, which avoids the deficiencies of filed-based metrological observation (Rhee et al., 2010; Rojas et al., 2011). The Normalized Difference Vegetation Index (NDVI), which can reflect the growth status of plants, has been widely used for evaluating the effects of drought on vegetation growth (Cunha et al., 2015; Klisch and Atzberger, 2016). Many NDVI-based drought indicators have also been developed, such as the Anomaly Vegetation Index (AVI) (Chen et al., 1994), the Temperature Vegetation Drought Index (TVDI) (Sandholt et al., 2002), and the Standardized Vegetation Index (SVI) (Peters et al., 2002). ET, an important component of terrestrial water cycles, is a more direct and effective indicator for reflecting ecosystem moisture status (Mu et al., 2007a, 2011). Remote sensing technology can provide spatially explicit ET information for terrestrial ecosystems (Jackson et al., 2005). Combining the MODIS-derived NDVI and evapotranspiration/potential evapotranspiration (ET/PET) data, Mu et al. (2013) developed a satellite-based drought index with fine resolution at the global scale. This index is known as Drought Severity Index (DSI). The DSI dataset has been proven to be capable of monitoring droughts over the last decade worldwide (Zhao and Running, 2011; Mu et al., 2013; Zhang and Yamaguchi, 2014).

The northwest regions of China and Inner Mongolia (NWIM), which are mainly situated in the northern inland of China, are characterized with a dry climate, scarce precipitation, intensive evaporation, and a fragile environment (Gou et al., 2015). The unique location and climate make these regions vulnerable to climate change and human disturbance. Previous research has demonstrated that Ningxia, Gansu, and Xinjing provinces have experienced different drought conditions in the past decade, and these conditions exerted great influence on environment and agricultural production (Gou et al., 2015; Huang et al., 2015; Wang et al., 2015; Zhang et al., 2016). However, most of these studies mainly focused on the regional scale. The extent and severity of drought occurrences as well as the effects of droughts on carbon and water cycles at the ecosystem scale are still open questions that require further study.

The first decade of the 21st century has been estimated as the warmest period since the 1880s. These extreme temperatures highlight the importance of identifying the drought condition for the dryland vegetation during this particular period (Zhao and Running, 2010). The primary objectives of this study are: (i) to examine the extent and duration of drought events in the forests and grasslands of the NWIM from 2000 to 2011 via remotely sensed DSI data; (ii) to quantify the annual changes of ecosystem-scale CUE and WUE for forests and grasslands during this period; and (iii) to explore the correlations between vegetation CUE, WUE, and climate variables to reveal the environment drivers of vegetation carbon and water utilization. The outcomes of this study will elucidate the extent and duration of drought occurrence in the forests and grasslands of the NWIM region, and the results provide guidance for the initiation of adaptation strategies to respond to the climate extremes.

## Materials and Methods

### Study Area

**FIGURE 1:**
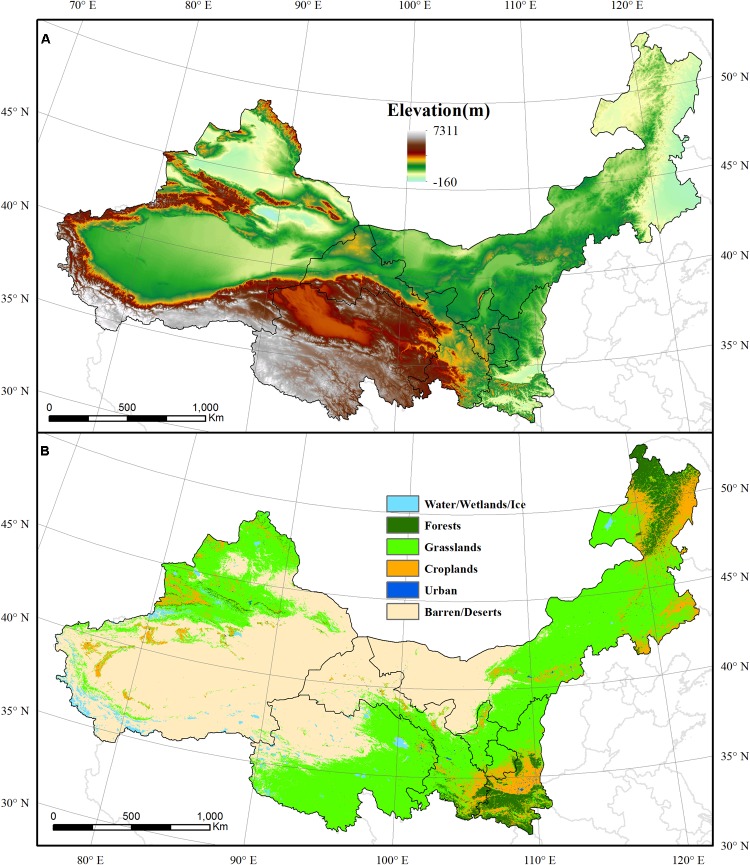
The elevation **(A)** and land use types **(B)** in the NWIM region.

The NWIM region covers three provinces (Shaanxi, Gansu, and Qinghai) and three autonomous regions (Ningxia Hui Autonomous Region, Inner Mongolia Autonomous Region, and Xinjiang Uygur Autonomous Region) between the latitudes of 37–53°N and the longitudes of 73–126°E (Figure 1). The total area of NWIM is approximately 4.15 × 10^6^ km^2^, occupying approximately 45% of the land area of China and supporting 10% of the population. Most of this region is characterized by arid and semi-arid climates with mean annual temperatures (MAT) ranging from −7 to 16°C. The mean annual precipitation (MAP) ranges from 10 to 1100 mm (Shi et al., 2007; Mu et al., 2013; Wang H. et al., 2013). The MAP exhibits an obvious latitudinal distribution. Xinjiang and northern Inner Mongolia exhibit lower values and the southern areas of Shaanxi, Gansu, and Qinghai present higher MAP values (Shi et al., 2007).

### Land Use and Land Cover Data

The land use and land cover map of the NWIM region was derived from the International Geosphere-Biosphere Project (IGBP) land cover dataset, which contains 17 land cover classes (Loveland et al., 2000). In this study, closed shrublands, open shrublands, woody savannas, savannas, and non-woody grasslands were regrouped as grasslands. The NWIM region has the largest typical steppe grassland in China, covering more than 41.00% of the total region. Evergreen needle forest, evergreen broadleaf forest, deciduous needle forest, and mixed forests were reclassified as forests, and these areas account for 4.70% of the total NWIM region. Barren/deserts, occupying 44.80% of the region, is the mostly widely distributed land use type in the NWIM region. Water bodies, croplands, and urban area made up to 1.52, 7.69, and 0.24% of total area, respectively. Only the grassland and forest areas were included in the following analyses.

### MODIS DSI, GPP, NPP, and ET Data

The annual MODIS DSI, GPP, NPP, and ET data from 2000 to 2011 for the NWIM region were obtained from the Numerical Terra dynamic Simulation Group at the University of Montana^1^. The DSI (0.05° spatial resolution) was based on the basis of ET/PET and snow-free growing season MODIS NDVI products for all vegetated land areas(Atkinson et al., 2011; Mu et al., 2013). The NDVI has been widely used to monitor the global vegetation photosynthetic activities due to its sensitivity to vegetation drought responses and associated water stress, especially in the water-limited regions (Huete et al., 2002; Justice et al., 2002). DSI is calculated as a standardized value, and its equation is expressed as follows:

(1) $${\mathrm{SA}}_{\text{NDVI}}=\frac{\mathrm{NDVI}-\bar{\mathrm{NDVI}}}{{\text{δ}}_{\text{NDVI}}}$$

(2) $${\mathrm{SA}}_{\text{EVA}}=\frac{(\mathrm{ET}/\mathrm{PET})-\bar{\left(\mathrm{ET}/\mathrm{PET}\right)}}{\delta_{\left(\text{ET}/\text{PET}\right)}}$$

(3) $$\mathrm{SA}={\mathrm{SA}}_{\text{NDVI}}+{\mathrm{SA}}_{\text{EVA}}$$

(4) $$\mathrm{DSI}=\frac{\mathrm{SA}-\bar{\mathrm{SA}}}{\delta_{\text{SA}}}$$

where *SA*_NDVI_, the standardized anomaly of the NDVI, is calculated using the long-term mean value of $\bar{\mathrm{NDVI}}$ and the standard deviation δ_NDVI_ during the period 2000–2011; *SA*_EVA_, the standardized anomaly of *ET/PET*, is calculated as the long-term mean value of $\bar{\mathrm{ET/PET}}$ and a standard deviation δ_(ET/PET)_; ET/PET means the ratio of ET to PET; *SA* is a sum of *SA*_NDVI_ and *SA*_EVA_; the DSI is a standardized anomaly of *SA*; $\bar{\mathrm{SA}}$ is the long-term mean value of *SA*_NDVI_ and *SA_(ET/PET)_*; δ_SA_ is the standardized deviation. The DSI value can be reclassified into 11 categories to indicate different drought conditions, which is shown in Table 1 (Mu et al., 2013; Zhang and Yamaguchi, 2014).

**Table 1:** The categories for drought conditions of the global DSI (Mu et al., 2013).

Category	Description	DSI
D5	Extremely drought	<−1.50
D4	Severe drought	−1.49 to −1.20
D3	Moderate drought	−1.99 to −0.9
D2	Mild drought	−0.89 to −0.60
D1	Incipient drought	−0.59 to −0.30
WD	Near normal	−0.29 to 0.29
W1	Incipient wet	0.30 to 0.59
W2	Slightly wet	0.60 to 0.89
W3	Moderately wet	0.90 to 1.19
W4	Very wet	1.20 to 1.50
W5	Extremely wet	>1.50

The new version of MODIS productivity products have been improved by matching the spatial resolution of metrological data to that of MODIS pixel, filling the missing value of FPAR/LAI data due to cloud contamination and malfunction of MODIS sensor, and updating the biome parameter look-up table according to the productivity data from flux tower measurements (Zhao et al., 2005; Gang et al., 2016a). GPP values are calculated as follows:

(5) $$\mathrm{GPP}=\varepsilon_{\max}\times 0.45\times \mathrm{SWrad}\times \mathrm{FPAR}\times \mathrm{fVPD}\times {\mathrm{fT}}_{\min}$$

where ε_max_ is the maximum light use efficiency under optimal conditions; *SW*_rad_ is the incoming short-wave solar radiation, of which 45% is Photosynthetically Active Radiation (PAR); FPAR is the fraction of PAR absorbed by the plant canopy; *fVPD* is vapor pressure deficits scalar, and *fT*_min_ is the daily minimum temperature (T_min_, °C) scalar.

NPP is calculated by subtracting the maintenance and growth respiration from GPP. It is calculated as follows:

(6) $$\mathrm{NPP}=\sum_{1}^{365}\mathrm{GPP}-R_{\text{m}\_\text{lr}}-R_{\text{m}\_\text{w}}-R_{\text{g}}$$

where *R*_m_lr_ is the maintenance respiration from living leaves and fine roots, and *R*_m_w_ is the annual maintenance respiration from living wood*, R_g_* is annual growth respiration. Detailed description for modeling MODIS GPP and NPP can be found in Zhao et al. (2005, 2006). Ecosystem-scale CUE for vegetation was calculated as the ratio of annual NPP to GPP in each grid.

The ET represents transpiration by vegetation and evaporation from canopy and soil surfaces. Based on the Penman-Monteith equation, the new version of MODIS ET dataset improved in many aspects, including the recalculation of the fraction of vegetation cover, the soil heat flux, and boundary layer resistance (Mu et al., 2007a,b, 2011). The ET algorithm is computed as follows:

(5) $$\lambda \;E=\lambda \;E_{\text{wet}\_\text{C}}+\lambda \;E_{\text{trans}}+\lambda \;E_{\text{SOIL}}$$

Where λ*E* is the total daily ET, λ*E*_wet_C_ refers to evaporation from the wet canopy surface, λ*E*_trans_ means the transpiration from the dry canopy surface, and λ*E*_SOIL_ is the evaporation from the soil surface (Mu et al., 2007a,b, 2011). The MODIS ET product has been validated and widely used in regional and global research (Mu et al., 2011; Gang et al., 2016a). The ecosystem-scale WUE for vegetation was calculated as the ratio of annual NPP to ET in each grid. The MODIS GPP, NPP, and ET performed reliable estimation accuracy on vegetation in northern China (Wang X. et al., 2013; Xiao, 2014; Liu Z. et al., 2015).

### Climate Factor Data

Meteorological data, including temperature and precipitation, from 2000 to 2011 were obtained from the China Meteorological Data Service Center^2^. The monthly data, interpolated by using ANUSPLIN, were used to generate the gridded MAT and MAP.

### Analysis of Temporal Dynamic

Equation 6 was used to quantify the linear trend of variables (including DSI, CUE, WUE, NPP, GPP, ET, MAT, and MAP) via the ordinary least square estimation:

(6) $$\mathrm{Slope}=\frac{n\times \sum_{\text{i}=1}^{n}i\times {\mathrm{Va}}_{\text{i}}-\left(\sum_{\text{i}=1}^{n}i\right)\left(\sum_{\text{i}=1}^{n}{\mathrm{Va}}_{\text{i}}\right)}{n\times \sum_{\text{i}=1}^{n}i^{2}-{\left(\sum_{\text{i}=1}^{n}i\right)}^{2}}$$

where *i* starts 1 for the year 2000, 2 for year 2001, and goes up to 12 for 2011; and *Va_i_* refers to the annual value of variable at time *i*, i = 1,….*n*, *n* = 12. The positive value of *Slope* in Eq. (6) indicates an increasing trend of variable, while the negative value connotes a decreasing trend during the 12-year period.

### Correlation Analysis of Climate Variables With Vegetation CUE and WUE

The correlations of vegetation CUE and WUE with climate variables, including DSI, MAP, and MAT, were calculated to reveal the controlling of climate variables that impact CUE and WUE. If the correlation coefficient passes the significance test, then an extremely significant (at 99% confidence level) or significant (at 95% confidence level) linear correlation is indicated.

## Results

### Drought Characteristics of Forest and Grassland in the NWIM Region

**FIGURE 2:**
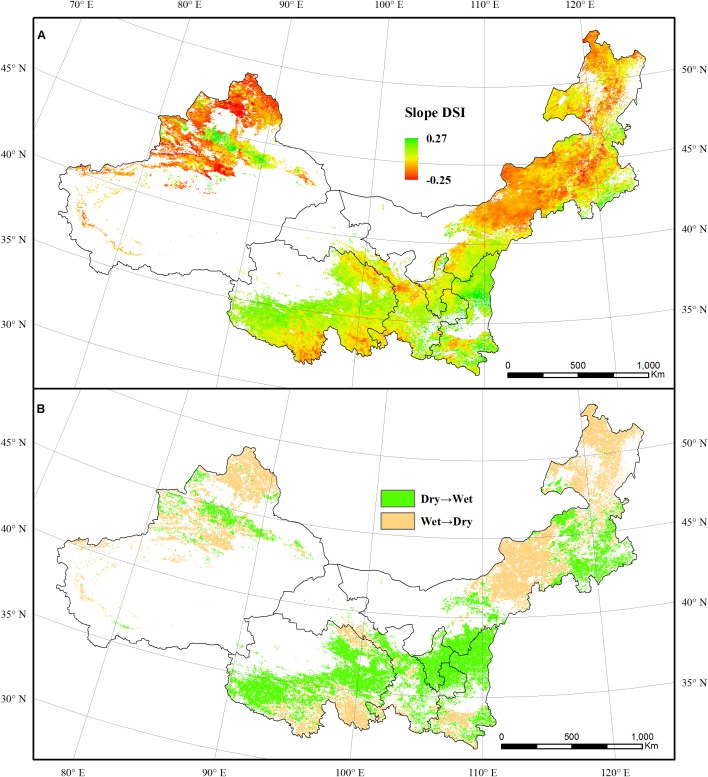
The spatial pattern of Drought Severity Index (DSI) dynamic **(A)**, the wetting and drying trend **(B)** of vegetation in the NWIM region from 2000 to 2011.

During the period of 2000–2011, the area of regions that became increasingly dry accounted for 49.90% of the total area of forest and grassland. These areas were mainly located in eastern Inner Mongolia and northern Xinjiang. The total area of regions that exhibited increasingly wetness was a little larger than the area exhibiting aridity. There regions with increasing wetness were mainly distributed within the 30–40°N, including the northern Shaanxi, Ningxia, Gansu, and Qinghai (Figure 2A). Nearly 3.73 × 10^4^ km^2^ of the forest regions that were dry in 2000 became wet in 2011. The areas under near normal conditions underwent the most obvious changes. Meanwhile, the forest regions that changed from wet to dry conditions reached an area of 15.56 × 10^4^ km^2^. This is far larger than the regions that became wet over the same period (Figure 2B). In contrast, 86.08 × 10^4^ km^2^ of the dry regions of grassland became wet during the 12-year period. Regions under D3 conditions were the largest contribution to this trend. The wet grassland area that became dry was relatively smaller, with the D2 condition making the largest contribution to such change.

**FIGURE 3:**
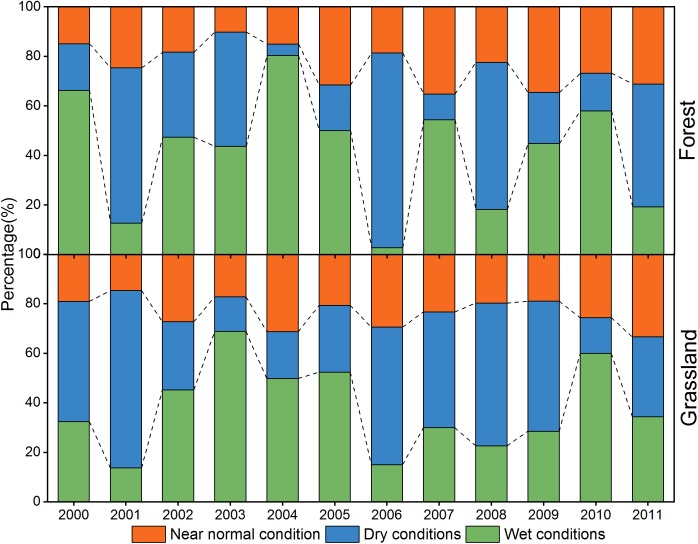
The percentage change of dry, wet, near normal conditions for forest and grassland during 2000–2011.

**FIGURE 4:**
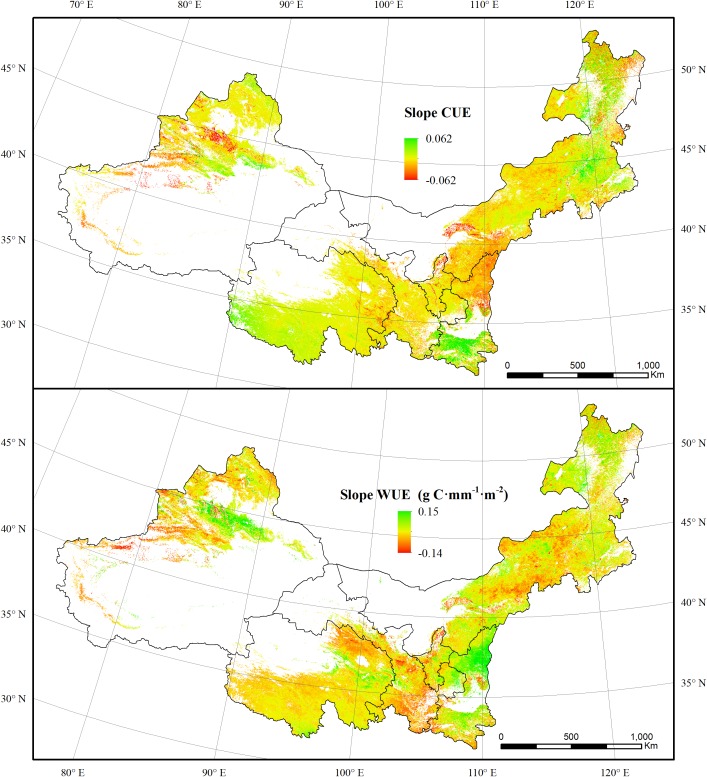
The spatial dynamics of CUE and WUE for forest and grassland from 2000 to 2011.

**FIGURE 5:**
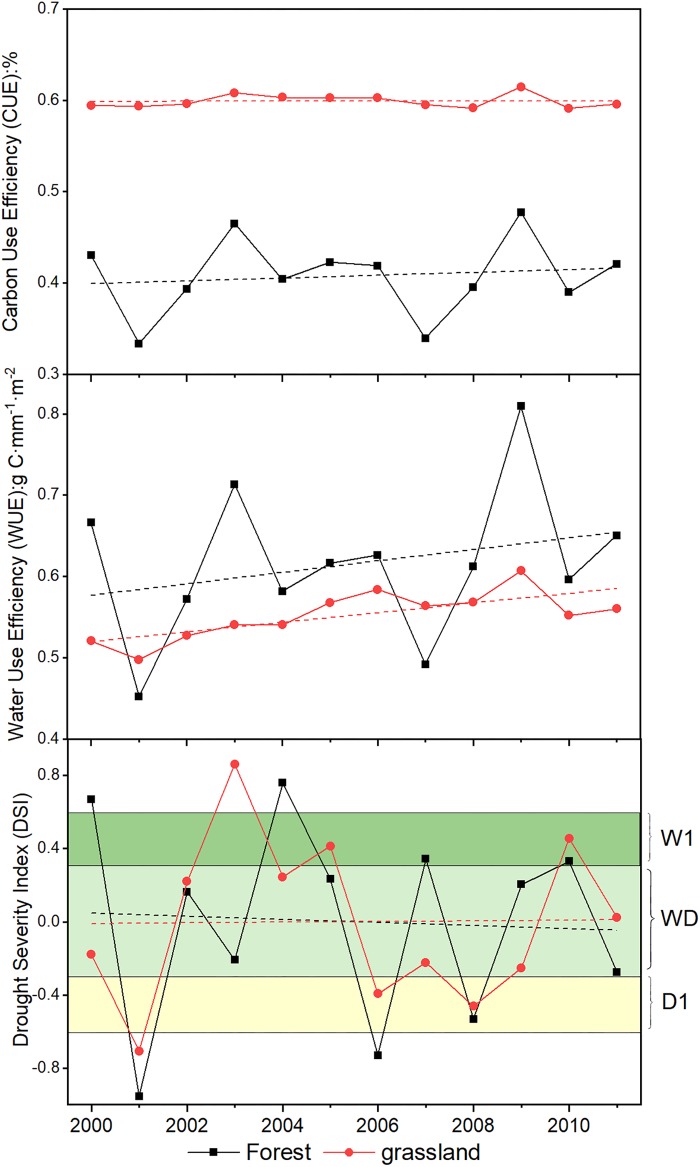
The temporal dynamics of CUE, WUE, and DSI for forest and grassland from 2000 to 2011.

**FIGURE 6:**
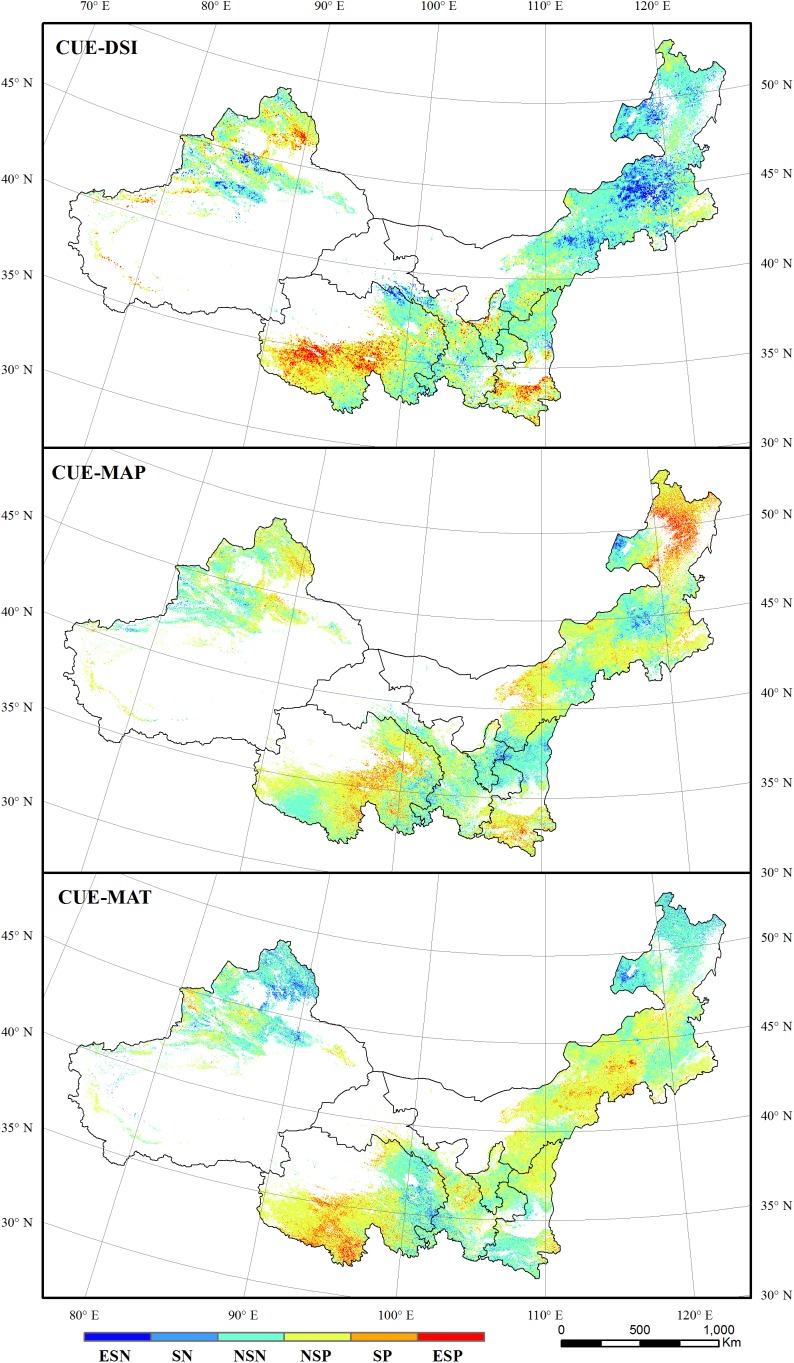
The correlations between grassland CUE and DSI, MAP, and MAT. ESN, extremely significant negative; SN, significant negative; NSN, non-significant negative; NSP, non-significant positive; SP, significant positive; ESP, extremely significant positive.

**FIGURE 7:**
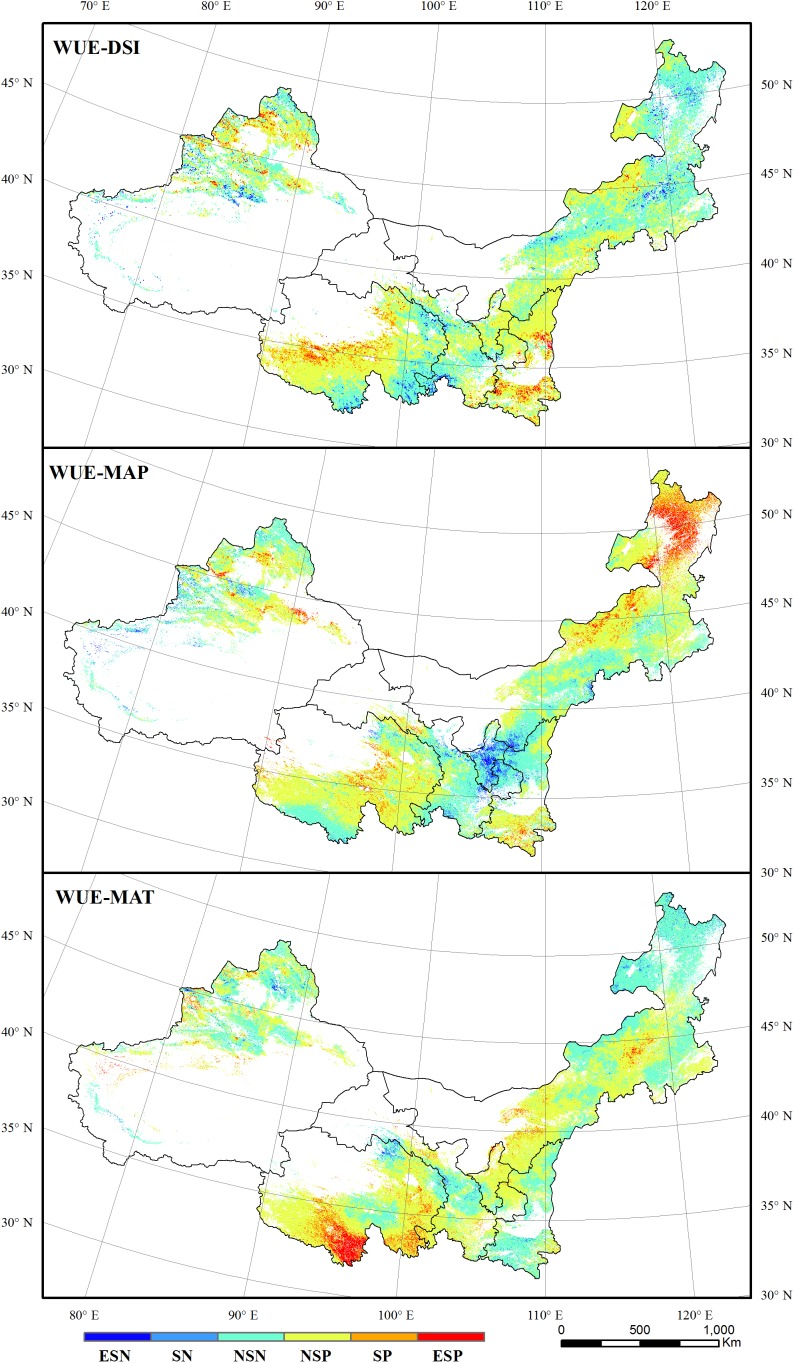
The correlations between grassland WUE and DSI, MAP, and MAT. ESN, extremely significant negative; SN, significant negative; NSN, non-significant negative; NSP, non-significant positive; SP, significant positive; ESP, extremely significant positive.

**FIGURE 8:**
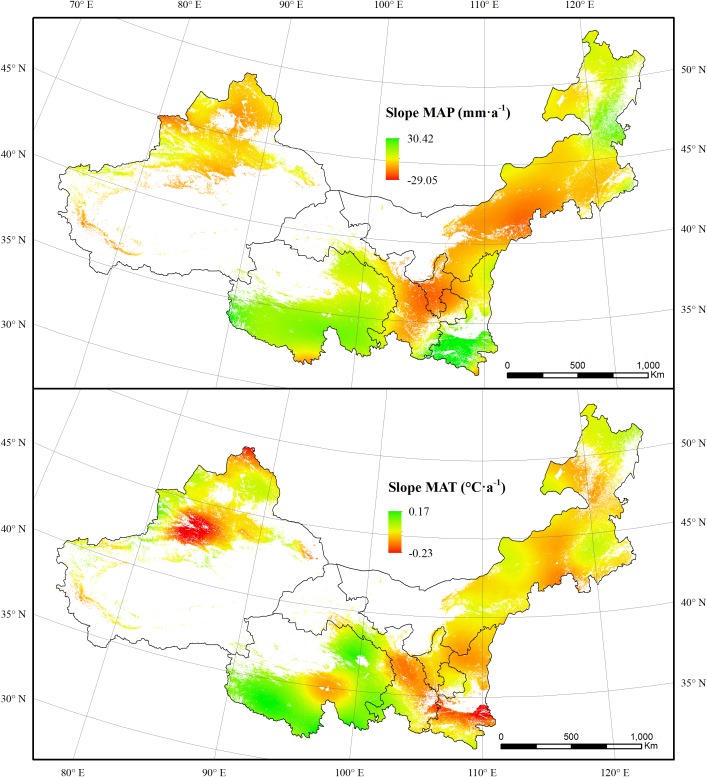
The spatial dynamics of MAP and MAT for forest and grassland from 2000 to 2011.

In 2001, 62.66% of forest regions were under dry conditions, and 38.90% were under extremely dry conditions (Figure 3). Meanwhile, 71.61% of grassland regions experienced drought, in which 32.51% were under D4 and D5 conditions. Most of forest and grassland regions were under wet conditions between 2002 and 2005. Both forest and grassland regions experienced an obvious drought in 2006. During 2006, 78.59% of the forest area and 55.49% of the grassland area were under dry conditions. Despite the widespread of dry conditions in 2006, most of regions were under D1-D3 conditions. After 2006, most of the forest regions were in wet conditions except in 2008 and 2011. Different drought levels occurred in most of the grassland regions until 2010. Overall, regions under wet conditions expanded gradually during the first half of the study period, and the dry spells expanded gradually in the grassland regions thereafter.

### The Spatiotemporal Dynamics of Vegetation CUE and WUE

The spatial dynamics of CUE and WUE for forest and grassland were firstly evaluated. 53.74% of forest and grassland regions showed an overall increasing trend of CUE over the entire study period, which mainly distributed in eastern Mongolia, and southern Shaanxi. Regions showing decreased CUE mainly occurred in the mid-west of Inner Mongolia, northern Shaanxi, and Tianshan Mountains region. In contrast, WUE increased in 85.98% of forest and grassland regions. Regions showing a decreased WUE mainly distributed in southern Gansu and northwestern Qinghai (Figure 4).

The annual CUE, WUE, and DSI during this period were plotted against time. The average CUE of grassland during the 12-year period was 0.60, higher than that of forest (0.41) for the same period (Figure 5). The value of grassland CUE remained steady over the past 12 years. WUE of forest exhibited fluctuating increase from 2000 to 2011, and the minimum and maximum values exhibited in 2001 and 2009, respectively. The range of change for grassland WUE was less drastic than that for forest. The WUE of grassland also peaked in 2009. The overall variation in forest and grassland DSI can be divided into three stages: the wetting trend from 2000 to 2004, the drying trend from 2004 to 2009, and the wet recovery from 2009 to 2011. The most severe drought occurred in 2001. Averagely, the forest and grassland experienced wet conditions in 3 years, while drought occurred in forest and grassland areas in 2001, 2006, and 2008. In all other years, forest and grassland regions were under near normal conditions. The spatiotemporal dynamics of GPP, NPP, and ET were presented in the Supplementary Material.

### The Controlling of Climatic Variables on Vegetation CUE and WUE

The correlations of vegetation CUE and WUE with DSI, MAP, and MAT were calculated to reveal the sensitivity of the vegetation carbon and water use to drought and climate variables. Regions with significant correlations (at 95 and 99% confidence level) between CUE and DSI amounted to 17.24% of the total study area. Area of forest regions that showed significant correlations (at 95 and 99% confidence level) between CUE and DSI reached 29.68% of the total forest regions, and 28.96% of this area exhibited positive correlations. For grassland, regions presenting significant positive and negative correlations between CUE and DSI accounted for 5.23 and 3.04% of total grassland area, respectively. Regions showing significant correlations (at 95 and 99% confidence level) between CUE and MAP accounted for 10.52% of the total area. These regions were mainly located in eastern Inner Mongolia, southern Shaanxi, and Qinghai (Figure 6). For forest, 15.20% of the area showed significant correlations between CUE and MAP, with 11.09% exhibiting positive correlations and 4.11% exhibiting negative correlation. In contrast, 17.47% of grassland regions showed significant correlations (at 95 and 99% confidence level) between CUE and MAP, in which 7.85% were positive correlations and 9.62% were negatively correlations. There were significant correlations between CUE and MAT in 7.79% of forest regions and 9.92% of grassland regions (at 95 and 99% confidence level), respectively. Grassland regions showing significant positive and negative correlations (at 95 and 99% confidence level) between CUE and MAT accounted for 5.99 and 3.94% of total grassland areas, respectively.

Regions with significant correlations (at 95 and 99% confidence level) between WUE and DSI, MAP, and MAT accounted for 10.37, 12.48, and 8.67% of the total study area, respectively. These regions were mainly located in eastern Inner Mongolia, Ningxia, and southern Qinghai (Figure 7). Regions that showed a significant correlation (at 95 and 99% confidence level) between WUE and DSI accounted for 15.89% of the forest area and 9.70% of the grassland area. The forest area with significant positive correlations (at 95 and 99% confidence level) between WUE and DSI was larger than the area with significant negative correlations (11.33% vs. 4.56%). Forest WUE was more correlated with MAP. Regions with significant correlations (at 95 and 99% confidence level) between WUE and MAP amounted to 35.68% of the total forest area, and 34.17% of these regions exhibited positive correlation. Forest WUE exhibited significant negative correlation (at 95 and 99% confidence level) with MAT over 4.23% of the forest area, and 1.02% of the forest region exhibited significant positive correlation. For grassland, regions showing significant positive correlations (at 95 and 99% confidence level) between WUE and DSI, MAP, and MAT were larger than those showing the negative correlations. WUE exhibited positive correlations with DSI, MAP, and MAT in 5.91, 5.53, and 7.58% of total grassland area, respectively, and showed negative correlations in 3.78, 4.13 and 1.52% of total grassland area, respectively.

## Discussion

In this study, the drought status and its impacts on dryland vegetation in northern China was evaluated by exploring the satellite-based data. Drought severity in forest and grassland as well as its influence on CUE and WUE at the ecosystem scale were investigated. During the period of 2000–2011, most of the forest and grassland in the NWIM region were in near normal and wet conditions. The widespread droughts mainly occurred in 2001 and 2006, and the drought in 2001 was the most severe across the entire study period. Previous studies have demonstrated that widespread and large droughts were frequently recorded in northern China since the late 1990s, and they intensified after 2000. The 2001 drought is considered to be one of the most severe droughts in terms of distribution and duration, which led to extensive agricultural and economical losses (Zhang et al., 2016). The strength of the East Asian summer monsoon in the developing and decaying phases of El Niño and La Niña events is probably one of the reasons that precipitated the recent drought in northern China (Yu et al., 2014).

Drought affects the vegetation carbon and water utilization mainly through influencing the photosynthesis and ET processes, particularly in regions where water supply is limited (Gang et al., 2016a). In this study, both the NPP and GPP for grassland increased during the 2000–2011 period. This synchronic changing pattern led to a steady state of grassland CUE during this period. In contrast, forest CUE was more sensitive to drought. The forest CUE presented a weak increasing trend with a curve similar to that of NPP. This was probably caused by the different sensitivities of forest NPP and GPP to the drought conditions (Zhang et al., 2014). In contrast, the rising NPP and decreasing ET both contributed to the overall ascending trend of WUE for forest and grassland. The WUE increased obviously in the Tianshan Mountains region and northern Shaanxi (Figure 4). The revegetation due to the “Grain for Green” project in the Loess Plateau has significantly increased the vegetation NPP during the past several decades (Xiao, 2014; Deng et al., 2017). The Tianshan Mountains region experienced a cooling trend, which probably is the leading reason for the WUE increase (Figure 8) (Guan et al., 2015). The canopy transpiration, net photosynthesis, and CO_2_ exchange were constrained by soil moisture (Mu et al., 2013). Soil moisture status affects the responses of heterotrophic respiration and photosynthesis to temperature. When experiencing a slight drought, the net photosynthesis rate of plants would decreases due to the reduced activity of ribulose diphosphate carboxylase and reduced photosynthetic capacity of mesophyll cells (Zhou et al., 2009). The widespread drought that occurred in 2006 did not lead to sudden drops in CUE and WUE values in 2006. However, there were drops in 2007. This indicates that the effects of drought might accumulate and appear in the following years. However, the photosynthetic organs of plants would be damaged under severe drought conditions. This would reduce plant water loss and photosynthesis (Arneth et al., 1998; Tian et al., 2009). This effect explains the obvious decreases of CUE and WUE in the 2001, when a widespread severe drought was recorded.

The correlation between vegetation WUE and DSI was relatively lower than the correlation between CUE and DSI. This implies that WUE was less sensitive to droughts than CUE for dryland vegetation in northern China. Drought affects the vegetation CUE mainly via the photosynthesis process (Gang et al., 2016b). Regions in eastern Inner Mongolia that are primarily vegetated by meadow showed significant negative correlations between CUE and DSI. The CUE increased in these regions despite a drying trend that was observed during 2000–2011. This increasing trend of CUE was probably caused by human interference, such as fencing or irrigation (Zhang et al., 2015; Yan et al., 2018). Regions showing significant positive correlations between CUE and DSI were mainly located in the northern fringe of Qinghai where became wet during the 12 years. The rising precipitation promotes the increase of both NPP and GPP in these regions (Bai et al., 2013). WUE can be affected by both the photosynthesis and ET process (Tian et al., 2010). Under slight and moderate drought conditions, the vegetation WUE would increase to mitigate the negative effects of water loss to some extent. This study found that area with significant positive correlations between WUE with MAP was larger than the area exhibiting significant positive correlations between WUE and DSI. This implies that rainfall more directly affected the vegetation WUE. Regions presenting the significant positive correlations between WUE and MAP were mainly distributed in eastern Inner Mongolia, Greater Khingan regions, where coniferous forests were mostly vegetated. Trees, such as *Larix gmelinii*, *Pinus tabulaeformis*, and *Abiescephalonica*, have higher WUE values because of low photosynthesis rates, ET rates, and stomatal conductance values (Lefi et al., 2004; Otieno et al., 2005). The photosynthesis ability can be maintained at a certain level even when a severe drought occurred (Maroco et al., 2000; Ogaya and Peñuelas, 2003; Lefi et al., 2004). Regions with a significant positive correlation between WUE and MAT were mainly distributed in the Three Rivers Source regions. This region is primarily characterized by alpine meadow. Temperature is reported as the primary factor affecting the growth of plants in this region (Xu et al., 2011; Guo et al., 2016).

## Conclusion

The dryland vegetation in northern China was deeply influenced by the drought during the 2000–2011 period, and forest and grassland reacted differently to drought conditions. Nearly half of the NWIM region became dry in 2000–2011. The areas of forest that experienced a drying trend was more than four times larger than the areas that became wet, whereas the area of grassland regions presenting a drying trend was closer to the area showing a wetting trend. The most widespread droughts occurred in 2001 and 2006, and the drought severity was higher in 2001 than 2006. In general, most of the vegetation was under wet conditions during the first half of the study period, and grassland subsequently experienced more frequent drought than forest. The forest CUE increased slightly, whereas the CUE of grassland remained steady over the 12-year period. Meanwhile, the decreased ET values of forest and grassland led to the overall increases in WUE values for forest and grassland. The DSI variation adequately explains the temporal dynamics of forest CUE and WUE over this period. In contrast, the CUE and WUE values for grassland were less sensitive to the recent drought conditions. Although there are a few uncertainties, our results suggest that the carbon and water use of forest in northern China suffered more from the recent droughts than that of grassland. Due to the data availability, only the 12 years of DSI and CUE, and WUE data were evaluated in this study. Given the complexity of drought events and the warming climate, it is important to continuously monitor various drought impacts on ecosystem-scale carbon and water use in dryland vegetation under future climate change.

## Data Availability

All datasets generated for this study are included in the manuscript and/or the Supplementary Files.

## Author Contributions

CG and ZW conceived and designed the research. CG, YZ, and MC collected the data and performed the research. LG, XG, and SP contributed data analysis and validation. CG wrote the manuscript.

## Conflict of Interest Statement

The authors declare that the research was conducted in the absence of any commercial or financial relationships that could be construed as a potential conflict of interest.

## Footnotes

1 http://www.ntsg.umt.edu/

2 http://data.cma.cn/
