# Supplementary material for: Drought-Induced Carbon and Water Use Efficiency Responses in Dryland Vegetation of Northern China
Source: Front Plant Sci. 2019 Feb 26;10:224. doi: 10.3389/fpls.2019.00224 (PMC6400040; doi:10.3389/fpls.2019.00224)
Supplement: Supplementary file 1 [file Table_1.DOCX]

Supplementary Material

## 1. Supplementary Figures


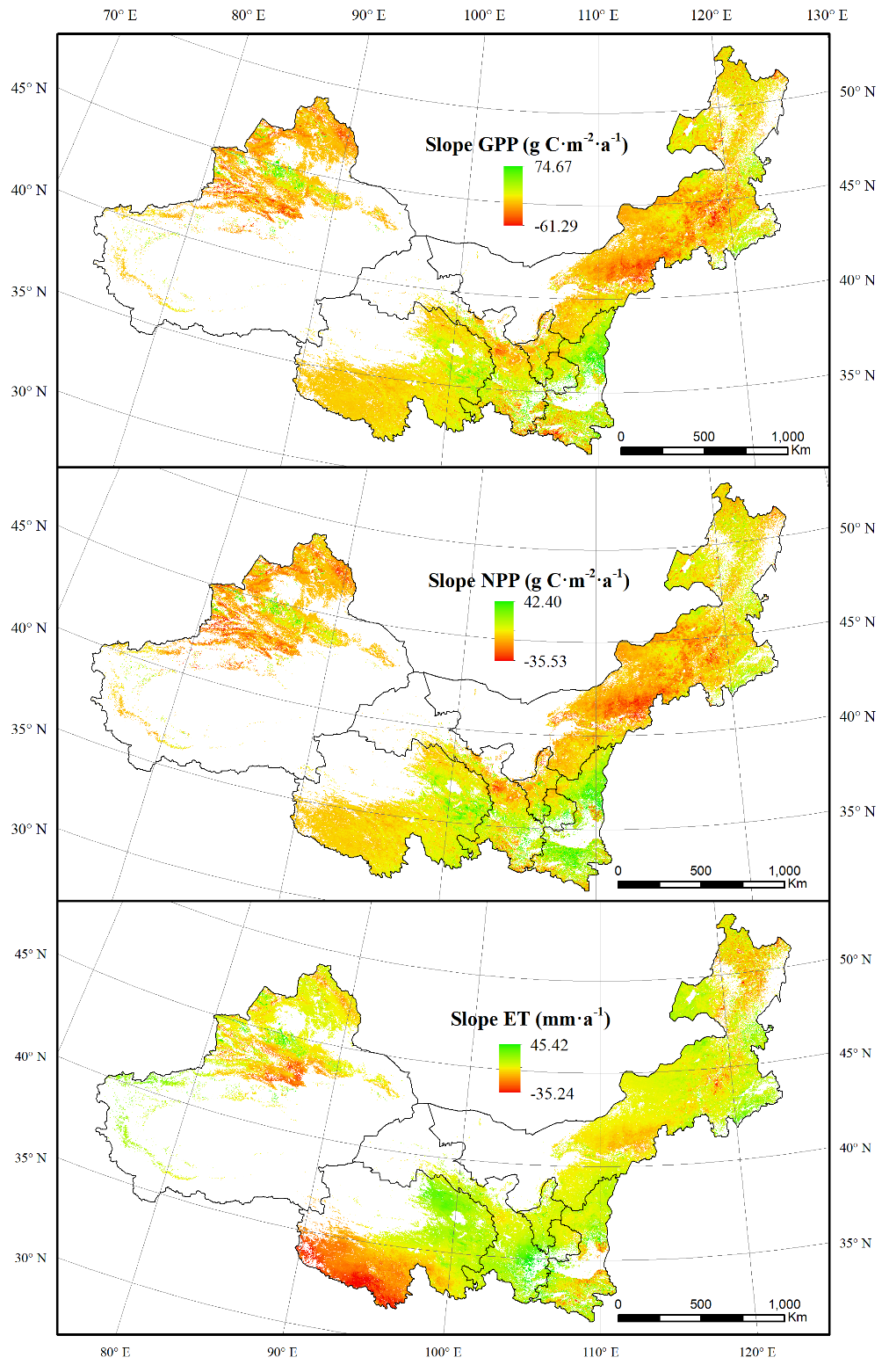


**Supplementary Figure 1.** The spatial dynamics of GPP, NPP and ET for forest and grassland from 2000 to 2011. Regions showing obvious increasing NPP and GPP are mainly distributed in Shaanxi, whereas regions presenting a decreased NPP and GPP mainly occur in Inner Mongolia and Xinjiang.


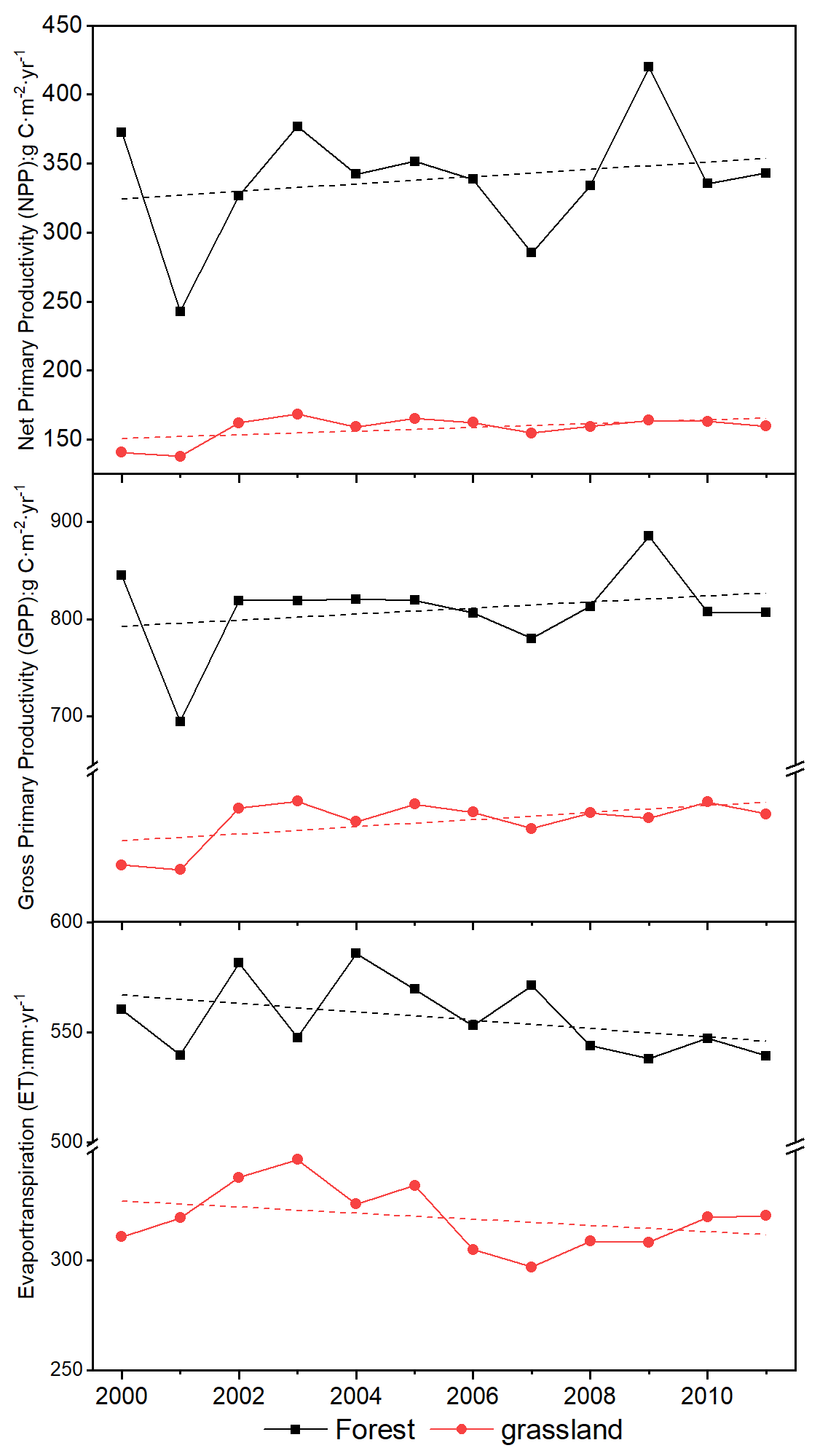


**Supplementary Figure 2.** The spatial dynamics of GPP, NPP and ET for forest and grassland from 2000 to 2011. NPP of forest and grassland shows an overall increasing trend during 2000-2011 period. GPP of forest and grassland presents a similar changing trend. By contrast, ET of forest and grassland decreased generally over the entire study period.
